# Coordinated Action of RTBV and RTSV Proteins Suppress Host RNA Silencing Machinery

**DOI:** 10.3390/microorganisms10020197

**Published:** 2022-01-18

**Authors:** Abhishek Anand, Malathi Pinninti, Anita Tripathi, Satendra Kumar Mangrauthia, Neeti Sanan-Mishra

**Affiliations:** 1 Plant RNAi Biology Group, International Center for Genetic Engineering and Biotechnology, New Delhi 110067, India; aanand@icgeb.res.in (A.A.); anita@icgeb.res.in (A.T.); 2 Biotechnology Section, ICAR-Indian Institute of Rice Research, Rajendranangar, Hyderabad 500030, India; malathi_amr@yahoo.co.in (M.P.); Satendra.KM@icar.gov.in (S.K.M.)

**Keywords:** RNA silencing, suppressors, virus disease, RTBV, RTSV, coat protein

## Abstract

RNA silencing is as an adaptive immune response in plants that limits the accumulation or spread of invading viruses. Successful virus infection entails countering the RNA silencing machinery for efficient replication and systemic spread in the host. The viruses encode proteins with the ability to suppress or block the host silencing mechanism, resulting in severe pathogenic symptoms and diseases. Tungro is a viral disease caused by a complex of two viruses and it provides an excellent system to understand the host and virus interactions during infection. It is known that Rice tungro bacilliform virus (RTBV) is the major determinant of the disease while Rice tungro spherical virus (RTSV) accentuates the symptoms. This study brings to focus the important role of RTBV ORF-IV in disease manifestation, by acting as both the victim and silencer of the RNA silencing pathway. The ORF-IV is a weak suppressor of the S-PTGS or stable silencing, but its suppression activity is augmented in the presence of specific RTSV proteins. Among these, RTBV ORF-IV and RTSV CP3 proteins interact with each other. This interaction may lead to the suppression of localized silencing as well as the spread of silencing in the host plants. The findings present a probable mechanistic glimpse of the requirement of the two viruses in enhancing tungro disease.

## 1. Introduction

Viruses represent the most invasive group of pathogens that are harmful for any host system they infect. Viral diseases in cultivated crops negatively affect plant morphology and physiology, thereby resulting in huge losses. However, resistance against viruses was observed as early as 1929 followed by observations of the phenomenon popularly known as “cross protection”. In this event, a plant infected with one virus showed resistance to the same or closely related virus on successive infection [1]. Subsequently it was shown that the plants experiencing disease symptoms on virus infection quickly recovered and the new tissues emerged without any symptoms [2]. These observations served as the early basis for engineering plants for viral resistance by integrating the viral genetic material into the plant genome and was termed as “pathogen derived resistance” (PDR) [2,3,4]. It could be later explained that cross protection and the recovery by PDR were associated with small RNA mediated gene silencing at the molecular level [5,6,7,8]. This phenomenon emerged as one of the most well-developed, robust, and potent defense strategies employed by plants and other eukaryotes [9,10,11].

The small RNA mediated gene silencing is a sequence-specific regulatory phenomenon resulting in mRNA degradation or translation inhibition at the post-transcriptional level and DNA methylation at the transcriptional level [10,12]. The process involves generation of 21 to 24 nt long double stranded RNA intermediates by the action of specific Dicer like (DCL) molecules that guide the silencing through Argonaute (AGO) containing protein complexes [11,13,14]. Depending on the mechanisms of biogenesis and function, the small RNAs are classified into microRNAs (miRNAs) and short interfering RNAs (siRNAs).

For successfully invading the hosts, viruses have co-evolved tools to effectively suppress the components of host silencing. The viral encoded suppressor molecules have been recognized as pathogenicity determinants as they have the ability to counteract antiviral silencing and play an important role in virulence [15,16,17]. The suppression activity has evolved independently such that existing viral proteins performing certain vital functions have gained an additional role of suppressing RNA silencing [18,19,20,21]. The Potyviral HC-Pro protein is the first and best described suppressor of host RNA silencing [22]. It is a multifunctional protein responsible for viral genome amplification, polyprotein processing, insect transmission, and long-distance movement. It also acts as a broad range pathogenicity enhancer by suppressing host silencing machinery [23,24]. The suppressor proteins do not share any co-evolutionary patterns among different viruses and there is very little chance of any significant homology or sequence similarity between them. Moreover, they have unique functional domains and properties, resulting in the distinctive mechanism of interacting with the components of host RNA silencing machinery [17,19]. All these facts add up to complicate their identification and classification. However, their ability to suppress RNA silencing, regardless of system or sequence specificity, has been exploited in excellent assays to characterize the suppressors. A number of in planta assays are available that are based on the suppression of the RNA silencing of a reporter transgene [25,26,27]. The principle behind these assays is that the silencing of a reporter transgene would be reversed by the effect of the suppressor when it is introduced and expressed in the system, resulting in expression of the reporter gene.

Tungro disease of rice is considered to be one of the most damaging viral diseases of rice prevalent in South and Southeast Asia and accounts for huge economic loss [28]. It is caused by a complex of two viruses, transmitted together by the green leafhoppers (GLH)—*Nephotettix virescens* and *N. nigropictus* [29,30]. The *Rice tungro spherical virus* (RTSV), a positive single strand RNA virus in the Secoviridae family [31], is required for transmission of the disease (Supplementary Figure S1). Single infection by RTSV does not produce any detectable symptoms of the disease. Its genome consists of 12433-nt RNA with a poly-A tail at the 3′ end that codes for a large polyprotein, which is cleaved to form three mature capsid proteins and other viral proteins [32]. *Rice tungro bacilliform virus* (RTBV), a double strand DNA pararetrovirus virus in the Caulimoviridae family (Supplementary Figure S1), is considered to be the major cause for the manifestation of the disease [33,34,35]. The RTBV genome contains four open reading frames (ORFs). The functions of ORF-I and ORF-II are yet not defined. The ORF-III codes for a large polyprotein, which is processed in to a movement protein (MP), a coat protein (CP), an aspartate protease (AP), and replicase proteins [33]. The ORF-IV transcript undergoes splicing to generate a protein that has a sequence motif similar to leucine zipper, although it lacks the DNA binding region. Though RTBV is the main determinant of disease symptoms, the symptoms get accentuated in the presence of RTSV [33,36]. Stunting, yellow orange discoloration of leaves and twisting of the leaf tips are the major symptoms in plants infected with virus [37,38].

It was demonstrated that transgenic rice plants expressing DNA encoding ORF-IV of RTBV, both in sense as well as in anti-sense orientation, were effective for controlling RTBV infection [39]. This study suggested that there is an involvement of small RNA molecules in providing resistance towards the disease. It was also reported that the rice silencing machinery responds to RTBV infection by generating typical viral siRNAs that are potentially associated with multiple AGOs in active RISC to direct silencing of the viral genes. However, RTBV appears to evade the repressive action of viral siRNAs by restricting their production to the non-coding region. It was also demonstrated that the protein encoded by ORF-IV of RTBV did not suppress cell autonomous silencing but suppressed the cell-to-cell spread of silencing [40].

The results obtained in this work suggest that the complexity of the disease is likely driven by the combined suppressor action of the two viral components of the tungro system. The ORF-IV protein of RTBV acts as a weak suppressor of RNA silencing, but its suppression activity is augmented in the presence of the RTSV proteins, protease, and coat protein 3. RTSV components might also have a possible role in enhancing the suppression of the cell-to-cell spread of silencing. The suppressor activities were assayed by utilizing an in planta assay based on the reversal of S-PTGS or pre-established stable GFP silencing [27]. The recognition of RTBV ORF-IV as a target and suppressor of the RNA silencing pathway has identified a key viral gene that can be exploited for boosting host plant resistance against tungro virus infection.

## 2. Materials and Methods

### 2.1. Plant Material

Rice leaf tissues were collected from healthy and tungro infected plants grown in controlled conditions at ICAR-Indian Institute of Rice Research, Hyderabad, India. To obtain tungro infected rice tissue, 15 days old plants of susceptible cultivar, Taichung Native 1 (TN1), were inoculated with the Hyderabad isolate of tungro virus using insect mediated virus transmission [38]. The insect vectors, green leafhoppers (GLH), were fed on tungro disease infected plants for 24 h (to acquire the virus particles) before releasing them onto healthy plants for 6 h. The disease symptoms were clearly observed after 15 days of virus inoculation. The presence of the virus was also confirmed by PCR using the methods described previously [41,42]. Thirty-day-old plants showing disease symptoms and virus presence were used for cDNA synthesis and small RNA library preparation.

### 2.2. Small RNA Library Sequencing and Computational Analysis

Samples from three different biological replicates were pooled and used for library preparation as per the manufacturer’s (Illumina) protocol. The libraries thus made were used for deep sequencing on the GAII sequencer, Illumina. This generated approx. 11 M tags per library, and data were delivered as sequences of 33 or 35 bases in length along with the base quality scores and read counts. The data have been deposited to the NCBI GEO database (SAMN17245506). The obtained raw sequence reads were processed computationally using in house developed pipelines to remove the putative miRNAs and predict siRNAs.

### 2.3. Plasmid Constructs

The individual ORFs of RTBV and RTSV were amplified from cDNA obtained from tungro-infected plants and cloned into pGEMTeasy vectors. The inserts were moved into binary vector pBI121 under the CaMV-35S promoter and NOS terminator. The transformants were analyzed by restriction analysis, PCR, and plasmid DNA sequencing. The recombinant plasmids (pBI121-ORF-IV, pBI121-ORF-I, pBI121-ORF-II, pBI121-ORF-Coat protein 3, pBI121-ORF-Polymerase, and pBI121-ORF-protease) were mobilized into *Agrobacterium tumefaciens* strains LBA4404 cells.

### 2.4. Generation of GFP Silenced Tobacco Plants and Agro-Infiltration

Agrobacterium-mediated reversal of GFP expression in GFP silenced *Nicotiana tabacum* L. cv. Xanthi leaves [27] was achieved through pressure infiltration, as described previously [43,44]. Briefly, wild type tobacco plants were transformed with the GFP gene constructs under CaMV35S promoter using agrobacterium cultures. The transformed plantlets were selected on a medium containing 100 mg/L Kanamycin. Screening for transgene integration was performed using genomic DNA PCR and Southern hybridization. The naturally silenced plants were screened by searching the transformed plants for the absence of GFP transcripts and GFP florescence. The naturally silenced plants were suitably tested and propagated.

Briefly, Agrobacterium culture was grown in YEM broth media at 28 °C, 180 rpm, until reaching an optical density of 1.0 at 600 nm (OD_600_). The culture was treated with 200 μM acetosyringone for 1 h prior to infiltration. The homogenous culture mixture was infiltrated in the young leaves with the help of a needleless syringe by generating a vacuum with the help of a finger on the dorsal side of the leaf and the mouth of the syringe on the ventral side. For co-agroinfiltrations, individual agrobacterium cultures were prepared until the OD reached 0.6 at 600 nm (OD_600_). Five ml of each culture at uniform OD values were mixed and centrifuged. The pellet was resuspended in minimum volume to obtain a homogenous suspension and treated with 200 µM acetosyringone (AS) for 1 h prior to infiltration in the young leaves.

Each experiment was performed in triplicates using 3–4 plants per set. For infiltrations, the third or fourth leaf from the top was used. Usually, multiple constructs were infiltrated in different sectors of each leaf to ensure uniformity. For molecular analysis, the infiltrated regions for the individual constructs were pooled. Each experiment was performed in triplicates to ensure repeatability.

### 2.5. cDNA Synthesis

First strand cDNA was prepared in 20 μL reactions from the total RNA isolated from the infiltrated leaf tissues, using 50 U of Super Script TM II reverse transcriptase (Invitrogen) and random hexamers according to the manufacturers’ protocol. The first strand cDNA of the total RNA was subjected to DNaseI treatment for 30 min.

### 2.6. Reverse Transcriptase Polymerase Chain Reaction (RT-PCR)

The semi-quantitative RT-PCR, for the amplification of GFP and suppressor genes (RTBV ORF-IV, RTBV ORF-I, and RTSV ORF-protease), was carried out using gene specific primers at initial sample denaturation at 95 °C for 5 min, followed by 25 cycles of strand separation at 94 °C for 1 min, annealing at 56 °C for 30 s, and extension at 72 °C for 30 s. The program was extended for 7 min at 72 °C. The tobacco 18S gene was used as a constitutive internal standard to evaluate cDNA content. The amplification products were analyzed on 0.8% agarose gel. The band intensities were quantified using the Alpha Imager Imaging System (Alpha Innotech, San Leandro, CA, USA).

### 2.7. Northern Blot Analysis

Total RNA was extracted from infiltrated regions of the leaves using the guanidium thiocynate extraction method [45]. A measure of 30 μg of total RNA from each plant sample was resolved on a formamide agarose gel to check for GFP transcript and small RNA. The RNA was transferred on Hybond N^+^ membranes (Amersham Pharmacia, Peapack, NJ, USA). Digoxigenin-11-dUTP (DIG) labelled DNA probes were generated via PCR labelling using the PCR DIG Probe Synthesis kit (Roche, Basel, Switzerland, catalogue No. 11636090910). A measure of 10 ng of purified plasmid DNA containing the full length GFP DNA was used as the PCR template and amplified with GFP forward (TCAAGGACGACGGGAACTACAAG) and reverse (GTGGTGGTGGCTAGCTTTGTA) primers. Northern blot analysis was performed at 50 °C using the protocol provided in the DIG Application Manual for Filter Hybridization (Roche). Following the hybridization, the blot was washed at 55 °C and probe detection was performed according to the manufacturers’ protocol using the DIG luminescent detection kit (Roche). The intensity of the individual bands was measured with respect to the background, and the integrated density value (IDV) was calculated using Alphaimager. Each experiment was performed in triplicates and the average values were used for plotting.

### 2.8. Yeast Two-Hybrid Analysis

Coding sequences of ORF-IV of RTBV and CP1, CP2, CP3, P1, polymerase, and protease of RTSV were cloned into pGBKT7 and pGADT7 vectors, respectively. Yeast two hybrid assays were performed using the Matchmaker Gold Yeast Two-Hybrid System (Clontech, Mountain View, CA, USA). The standard protocol given in the kit was followed for the Y2H assay. The yeast growth media (YPD medium, Clontech), SD growth medium (Ade^−^ His^−^ Leu^−^ Trp^−^), and supplement media (Clontech) were used for the Y2H experiment.

## 3. Results

### 3.1. ORF-IV of RTBV Is a Potential Hotspot for siRNA Generation

**Figure 1:**
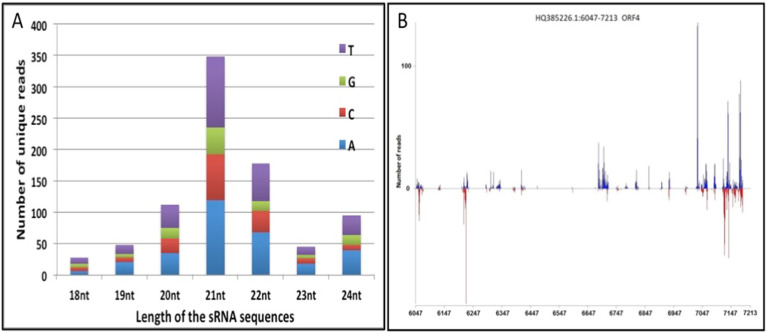
Size distribution and mapping of small RNA reads obtained from tungro infected data sets: (**A**) Length distribution of the small RNA sequences showing the distribution of the first nucleotide in the molecules. (**B**) Plot of siRNA molecules mapping of ORF-IV for RTBV. The nucleotide position of ORF is shown on the X-axis. siRNAs mapping to the positive strand of the ORF are represented by blue lines, while those mapping to the negative strand are represented by red lines.

The small RNA sequencing data sets from tungro-infected rice leaves (TL) were analyzed using in house developed pipelines to predict and map the siRNAs to the RTBV genome. The small RNA reads were aligned to the RTBV genome sequence (Accession no. NC_001914.1), using a Bowtie tool, allowing for only one mismatch at either the first or last nucleotide. The TL small RNA libraries contained a total number of 2,885,141 reads, representing 554,305 unique sequences. Read length distribution analysis showed that the 21-nt small RNAs, majorly representing the canonical miRNAs and siRNAs, were the highest in number, indicating a large diversity in their sequences (Figure 1A). The first nucleotide of these molecules was either A or T in 68% of molecules (Figure 1A), as shown in previous studies of rice small RNAs [40,46,47,48].

After removing the sequences mapping to the rice genome, the remaining sequences were aligned to the RTBV ORFs. This analysis identified 1690 unique reads that were perfectly aligned (with zero mismatch) at different positions on ORFs of both the strands of the RTBV (Supplementary Figure S2). It was observed that almost 50% (850) of the unique reads aligned at different positions distributed over most of the ORF-IV region, although in terms of total numbers, the majority of siRNAs were clustered towards its 3′ end. The abundance of siRNA biasing could be observed more on the positive strand of the ORF than on the negative strand (Figure 1B). Analysis of the mapping pattern and the abundance of the putative siRNAs on RTBV ORFs showed that ORF-IV acted as a probable hotspot for small RNA generation (Figure 1B) in the infected leaf tissues.

### 3.2. ORF-IV Can Suppress Pre-Established RNA Silencing

**Figure 2:**
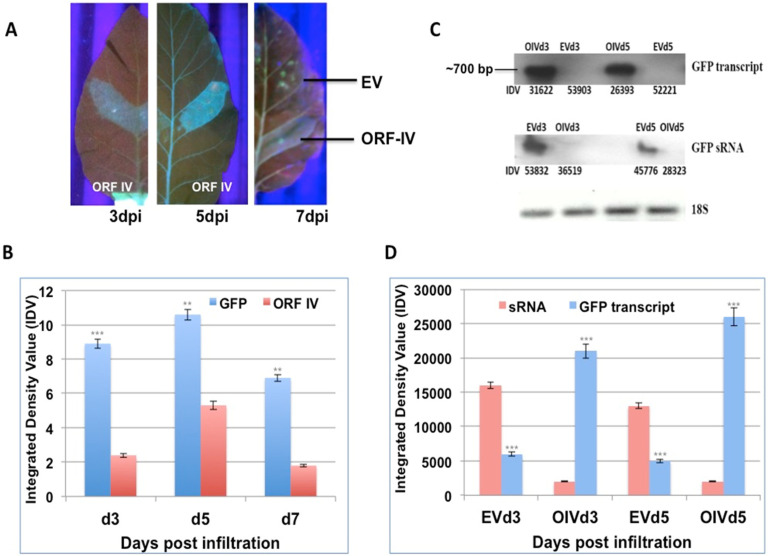
Reversal of GFP silencing by RTBV ORF-IV and its molecular analysis. (**A**) The different panels exhibit GFP fluorescence in the region infiltrated with ORF-IV at 3, 5, and 7 days post infiltration (dpi). The region infiltrated with the empty vector (EV) served as the control. (**B**) The graph represents the normalized Integrated Density Values (IDV) for the GFP and ORF-IV transcripts relative to 18S control cDNA at 3 dpi (d3), 5 dpi (d5), and 7 dpi (d7). (**C**) Northern blots of GFP transcript and GFP sRNA in regions infiltrated with ORF-IV (OIV) and EV. The 18S cDNA band served as the control. (**D**) The graph represents the normalized values of GFP transcripts and GFP siRNA as measured in the infiltrated regions at 3 dpi and 5 dpi. **, *** Significance at probability levels of 1% and 0.1%, respectively (ANOVA single factor).

Leaves of stably silent GFP tobacco lines [27] were infiltrated on one side of the midrib with Agrobacterium cultures containing the individual ORFs of RTBV (ORF-I, ORF-II, and ORF-IV) and RTSV (protease, polymerase, and coat protein 3). Leaves were collected at 3, 5, and 7 days post infiltration (dpi) and scanned under UV-light to observe for GFP fluorescence, as proof for the reversal of silencing by the infiltrated suppressor. The RTBV ORF-I, ORF-II, and the RTSV ORFs did not show clear suppressor activity in individual infiltrations (Supplementary Figure S3). In regions infiltrated with RTBV ORF-IV, a low level of fluorescence was observed in leaves at 3 dpi, while significant fluorescence could be observed at 5 dpi, and this dipped again by 7 dpi (Figure 2A). No fluorescence was detected in the regions infiltrated with empty vector that served as the control. This showed that ORF-IV acts as a weak suppressor of the S-PTGS or pre-established stable silencing.

The suppressor activity was confirmed by validating the levels of the GFP transcript and GFP small RNAs using northern blot (Figure 2B). It was seen that GFP transcripts accumulated only in the regions where ORF-IV was transcribed, indicating its interference with the silencing machinery. The time kinetics revealed that the GFP transcript started forming by 3 dpi, even though significant florescence levels could be detected at 5 dpi (Figure 2C). Correspondingly, low levels of GFP small RNAs were observed in leaf regions infiltrated with ORF-IV, while prominent levels of the GFP small RNAs were detected in the empty vector infiltrated leaf sections (Figure 2B,D). The negative correlation between the small RNA and ORF-IV transcript accumulation confirmed the suppressor action of ORF-IV.

### 3.3. RTSV Coat Protein Enhances the Suppressor Activity of ORF-IV

**Figure 3:**
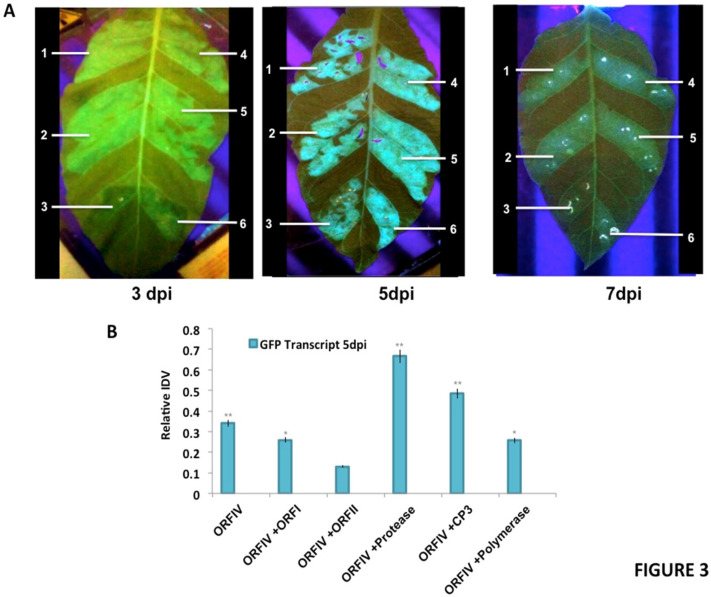
Reversal of GFP silencing by co-infiltration of RTBV and RTSV ORFs. (**A**) The different panels exhibit representative pictures to show GFP fluorescence in the infiltrated regions at 3, 5, and 7 days post infiltration (dpi). The markings indicate the region infiltrated with (1) ORF-IV construct, (2) ORF-IV co-infiltrated with RTBV-ORF-I, (3) ORF-IV co-infiltrated with RTBV ORF-II, (4) ORF-IV co-infiltrated with RTSV ORF coding for coat protein 3, (5) ORF-IV co-infiltrated with RTSV ORF coding for protease, and (6) ORF-IV co-infiltrated with RTSV ORF coding for polymerase. (**B**) Normalized values for GFP transcripts in the different infiltrated regions at 5 dpi, confirmed using RT PCR. *, ** Significance at probability levels of 5% and 1%, respectively (ANOVA single factor).

It is well known that tungro disease is manifested by the co-infection of RTBV and RTSV. The presence of RTBV alone causes mild disease symptoms, but these are accentuated in the presence of RTSV [33,36]. Thus, it was important to further investigate the nature of the RNA silencing suppression activity of RTBV ORF-IV in the presence of RTSV. For this, the same assay system was employed and the leaves were infiltrated, respectively, with ORF-I and ORF-II of RTBV and RTSV ORF encoding protease, coat protein 3 (CP3), and polymerase as co-infiltrations with the ORF-IV (Figure 3). The infiltrations with ORF-IV alone served as the reference point for assaying the suppressor activity.

It was observed that at 3 dpi, enhanced suppressor activity is observed in regions co-infiltrated with ORF-IV and RTSV ORF, encoding CP3 and polymerase. At 5 dpi, the suppression activity was significantly enhanced in individual co-infiltrations with RTSV ORFs, but decreased in the presence of the RTBV ORF-II. The enhanced suppression activities in the regions co-infiltrated with RTBV ORF-IV and RTSV ORFs encoding protease as well as CP3 were sustained up to 7 dpi (Figure 3A). To validate the results, molecular analysis of the co-infiltrated zones was performed to check for the presence of GFP as well as the ORF transcripts. For each transcript, the relative band intensity values were calculated by normalizing with respect to the 18S control. Normalized values for GFP transcripts with respect to ORFIV in the different infiltrated regions at 5 dpi were plotted (Figure 3B). A higher level of expression was seen for GFP in the regions where ORF-IV was co-infiltrated with RTSV ORFs coding for protease and CP3, indicating their role in the enhancement of the suppression activity encoded by RTBV. This clearly demonstrates that the early onset of suppression of ORF-IV is enhanced and sustained by the activity of RTSV ORFs, which may potentially lead to a potent infection in the rice plant. It was also observed that, at 3 dpi, the respective presence of RTSV protease and CP3 caused a transient spread of silencing beyond the infiltrated zone (Figure 3A). This indicates the suppression of both the local siRNAs as well as the mobile signals responsible for the systemic spread of silencing; however, further investigation is required to understand the mechanism of action.

### 3.4. RTBV ORF-IV Interacts with RTSV CP3 Protein

**Figure 4:**
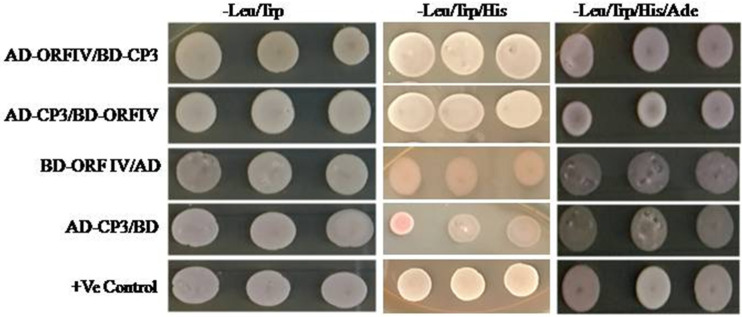
Yeast two-hybrid assay for direct interaction study. Yeast colonies of pGAD-CP3 and pGBD-ORF-IV were co-transformed and selected on (i) two drop out plate (Leu^−^ Trp^−^), (ii) triple drop out (Leu^−^ Trp^−^ His^−^), and (iii) triple drop out (Leu^−^ Trp^−^ His^−^) supplemented with 5 μ M 3-AT. Co-infiltration of pGAD-CP3 with pGBD and pGAD with pGBD-ORF-IV was performed as the control.

To confirm if enhancement in ORF-IV suppressor activity by RTSV proteins involves their direct interaction, yeast two hybrid (Y2H) assay was performed. Yeast cells co-expressing binding domain (BD) fused with ORF-IV and activation domain (AD) fused with CP3 could grow on both three-drop-out media (without amino acids Leu: leucine, TRP: tryptophan, and His: histidine) and four-drop-out media (without amino acids: Leu, TRP, His, and Ade: adenine). The results show a direct and strong interaction of RTBV ORF-IV with RTSV CP3 (Figure 4 and Supplementary Figure S4). The other proteins of RTSV coding for CP1, CP2, P1, polymerase, and protease did not show any interaction with RTBV ORF-IV.

## 4. Discussion

The host RNA silencing mechanisms are triggered in response to viral infection to produce siRNAs as a first line of defense [49,50,51,52]. The siRNAs act by directing the cleavage of the complementary viral transcripts or by inducing transcriptional silencing. During virus disease, the precursors of viral siRNAs are possibly produced by Pol II-mediated bi-directional transcription of the viral DNA [53,54,55]. The viral double stranded RNAs can be processed by DCL2 and DCL3 to generate the 22-nt and 24-nt siRNAs, respectively, whereas DCL1 and DCL4 process 21-nt siRNAs [56]. The 21-nt species become associated with AGO1 proteins to direct post-transcriptional gene silencing in order to allow an immediate recognition and elimination of incoming viruses [57,58,59,60], while 24-nt siRNAs associate with AGO4 to direct cytosine methylation of complementary target DNA [46,47,48] and are responsible for the systemic spread of silencing.

Analysis of small RNA read length distribution showed that the 21-nt small RNAs represent the major group. This is in confirmation with an earlier analysis of the small RNA libraries prepared from tungro-infected leaves [40]. It has been reported that the maximum production of abundant viral siRNAs was restricted to the 599-bp noncoding region adjacent to the Pol II transcription start site. We observed that amongst the unique small RNA reads that aligned to the RTBV ORFs, 50% alignment was on the ORF-IV region with major clustering towards its 3′ end. The ORF-IV transcript arises by splicing and joining the coding sequences to the 5′ untranslated region [61]. It has been demonstrated that, in DNA viruses, the intergenic regions harboring bidirectional promoter elements between the transcription start sites are a poor source of viral siRNAs [54,62], while the transcripts of either polarity are preferential sources of double strand RNA, which are subsequently processed by the DCLs [63].

The in planta silencing suppression assay was based on screening for the reversal of GFP silencing in stably silenced tobacco plants. High levels of transgenic RNA are recognized as aberrant by the rate limiting cellular cofactors, and this triggers its conversion into a duplex by the host RNA dependent RNA polymerase [64]. The dsRNA initiates siRNA production, which in turn leads to cleavage of the transcripts called sense PTGS (S-PTGS). Both DCL4- and DCL2-processed secondary siRNAs were reported to be effective in silencing activity when an inducing transgene is expressed constitutively. At later stages, the siRNAs can guide the protein complexes to switch off gene expression, thereby establishing the stable silenced state for the gene [65,66,67]. This theory is substantiated by the frequent induction of silencing by over-expression of the sense gene(s) [68,69]. The transgene-induced and virus-induced gene silencing overlap mechanistically, so the process has been exploited to identify the suppressors of RNA silencing [27] by its action of reversing the inhibition of GFP expression.

The ORF-IV demonstrated suppression RNA silencing activity in planta (Figure 2). It has been reported that RTBV protein encoded by ORF-IV may not be essential for viral replication, assembly, or movement, as other pararetroviruses belonging to the family Caulimoviridae do not possess any related gene. This locus is a hub for siRNA generation, so it is likely to have evolved the ability to counteract the host RNA silencing defenses [70]. An indication for this was provided by GFP co-infiltration experiments wherein ORF-IV was shown to suppress the silencing signal in boundaries of infiltrated zones, even though it was reported not to suppress cell autonomous silencing [40]. However, in this study, we identified ORF-IV as a weak suppressor of the S-PTGS or pre-established stable silencing, and the results were supported by molecular validations.

It has been reported that RTSV individual infection does not cause any major disease symptoms, but in the presence of RTBV, the severity of infection increases. RTSV is mainly known to promote the transmission of the disease. Our results demonstrate that specific RTSV proteins may help in early onset of suppression by ORF-IV and in the sustenance of the activity, which potentially leads to a potent infection in the rice plant. It should be noted that both RTBV and RTSV are required to produce typical viral symptoms of tungro disease [33]. The results of co-infiltration experiments showed that the suppression activity of ORF-IV was enhanced in the presence of RTSV ORF-protease and CP3 (Figure 3). It was also observed that the presence of RTSV protease and CP3 caused a transient spread of the suppression of silencing beyond the infiltrated zone. These observations are supported by the report that RNAi based silencing of the CP3 gene caused a restraint of the tungro disease in rice [71]. The important role of RTBV ORF-IV and RTSV CP3 in virus diversification and evolution has been suggested [34,72].

Yeast based interaction studies suggested that RTSV ORF-IV and RTBV CP3 proteins might act together in suppressing the silencing pathway (Figure 4). However, the nature of protein interactions in vivo and their association in suppressing the RNAi driven host defense need to be confirmed by co-immunoprecipitation assays and other studies. No interaction was observed between RTSV protease and RTBV ORF-IV, though silencing suppression was enhanced in their co-presence. This observation indicated that the two proteins might be suppressing the silencing pathway by either targeting different components or acting independently on a common intermediate of the silencing pathway. Further work will, therefore, be required to unravel their mechanistic insights.

## 5. Conclusions

This study brings to focus the important role of RTBV ORF-IV in disease manifestation. This locus acts as both the victim and silencer of the RNA silencing pathway in host plants, representing a key target that can be exploited for boosting host plant resistance against tungro virus infection. The study also provides evidence of the complementation of RNA suppression activity to RTBV ORF-IV by RTSV CP3 and the direct interaction between the two proteins. This implies the necessity of the presence of both of the viruses to cause the disease. It can thus be hypothesized that RTBV encodes suppression activity for handling the localized silencing activity of the host plant, whereas the RTSV components help in enhancing the suppression of the cell-to-cell spread of silencing, thus sustaining the spread of infection. The silent role of RTSV CP3 to enhance and extend the suppression activity of ORF-IV indicates that the nature and degree of interaction of the viral proteins with each other in the host need to be further elucidated to understand the complexity of tungro disease manifestation and its protraction. The results obtained in this study provide a clear indication on the complexity of tungro disease driven by the synergy of two disparate viruses.

## Data Availability

Not applicable.

## Supplementary Materials

The following are available online at https://www.mdpi.com/article/10.3390/microorganisms10020197/s1, Figure S1: Genomic organization and structure of RTBV and RTSV. Figure S2: Plot of siRNA molecules mapping to different positions on both the strands of the RTBV genome. The boxes on top show the mapping pattern on individual RTBV ORFs. The nucleotide positions are indicated on the scale in each figure. The siRNAs mapping to positive strand of the ORF are represented by blue lines while those mapping to the negative strand are represented by red lines. Figure S3: Individual infiltrations to assay the in planta reversal of GFP silencing activity by different RTBV and RTSV ORFs. The RTBV ORF-I, ORF-II and the RTSV ORFs encoding protease, polymerase and coat protein 3 did not show a clear suppressor activity. The region infiltrated with empty vector (EV) served as control. Figure S4: Plates showing the yeast two-hybrid interaction of pGAD-CP3 and pGBD-ORF-IV. Yeast colonies were co-transformed and selected on (A) two drop out medium (Leu^−^ Trp^−^) and (ii) four drop out medium (Leu^−^ Trp^−^ His^−^, Ade^−^). Co-infiltration of pGAD-CP3 with pGBD and pGAD with pGBD-ORF-IV was performed as control. The positive control was same as that provided in the kit. For negative control empty AD and BD vectors were co-transformed.

## References

[1] McKinney H.H. (1929). Mosaic diseases of the Canary Islands; West Africa and Gibraltar. J. Agri. Res..

[2] Lindbo J.A., Silva-Rosales L., Proebsting W.M., Dougherty W.G. (1993). Induction of a highly specific antiviral state in transgenic plants: Implications for regulation of gene expression and virus resistance. Plant Cell.

[3] Beachy R.N. (1997). Mechanisms and applications of pathogen-derived resistance in transgenic plants. Curr. Opin. Biotechnol..

[4] Kyrychenko A.M., Kovalenko O.G. (2018). Basic engineering strategies for virus-resistant plants. Cytol. Genet..

[5] Ratcliff F., Harrison B.D., Baulcombe D.C. (1997). A similarity between viral defense and gene silencing in plants. Science.

[6] Garcia-Ruiz H. (2019). Host factors against plant viruses. Mol. Plant Pathol..

[7] Ramesh S.V., Yogindran S., Gnanasekaran P., Chakraborty S., Winter S., Pappu H. (2021). RVirus and viroid-derived small RNAs as modulators of host gene expression: Molecular insights into pathogenesis. Front. Microbiol..

[8] Teixeira R.M., Ferreira M.A., Raimundo G., Fontes E. (2021). Geminiviral triggers and suppressors of plant antiviral immunity. Microorganisms.

[9] Ding S.W. (2010). RNA-based antiviral immunity. Nat. Rev. Immunol..

[10] Prasad A., Sharma N., Muthamilarasan M., Rana S., Prasad M. (2019). Recent advances in small RNA mediated plant-virus interactions. Crit. Rev. Biotechnol..

[11] Sanan-Mishra N., Jailani A.A.K., Mandal B., Mukherjee S.K. (2021). Secondary siRNAs in plants: Biosynthesis; various functions; applications in virology. Front. Plant Sci..

[12] Sanan-Mishra N., Mukherjee S.K. (2007). A peep into Plant miRNA world. Open Plant Sci. J..

[13] Pelaez P., Sanchez F. (2013). Small RNAs in plant defense responses during viral and bacterial interactions: Similarities and differences. Front. Plant Sci..

[14] Sinha V., Anand A., Mukherjee S.K., Sanan-Mishra N., Datta A., Fakruddin M., Iqbal H.M.N., Abraham J. (2017). RNAi based strategies for enhancing plant resistance to virus infection. Advances in Biotechnology.

[15] Qu F., Morris T.J. (2005). Suppressors of RNA silencing encoded by plant viruses and their role in viral infections. FEBS Lett..

[16] Anand A., Mukherjee S.K., Sanan-Mishra N., Mendez-Vilas A. (2013). Tools for pathogenicity: Virus encoded RNA silencing suppressors. Recent Microbial Pathogens and Strategies for Combating Them: Science, Technology and Education.

[17] Sanan-Mishra N., Chakraborty S., Gupta D., Mukherjee S.K., Rajewsky N., Jurga S., Barciszewski J. (2017). RNAi suppressors: Biology and mechanisms. Plant Epigenetics; RNA Technologies.

[18] Carrington J.C., Kasschau K.D., Johansen L.K. (2001). Activation and suppression of RNA silencing by plant viruses. Virology.

[19] Silhavy D., Molnar A., Lucioli A., Szittya G., Hornyik C., Tavazza M., Burgyan J. (2002). A viral protein suppresses RNA silencing and binds silencing-generated; 21- to 25-nucleotide double-stranded RNAs. EMBO J..

[20] Kumar V., Mishra S.K., Rehman J., Taneja J., Sundaresan G., Sanan-Mishra N., Mukherjee S.K. (2015). Mungbean yellow mosaic Indian virus encoded AC2 protein suppresses RNA silencing by inhibiting Arabidopsis RDR6 and AGO1 activities. Virology.

[21] Veluthambi K., Sunitha S. (2021). Targets and mechanisms of geminivirus silencing suppressor protein AC2. Front. Microbiol..

[22] Anandalakshmi R., Pruss G.J., Ge X., Marathe R., Mallory A.C., Smith T.H., Vance V.B. (1998). A viral suppressor of gene silencing in plants. Proc. Natl. Acad. Sci. USA.

[23] Pruss G., Ge X., Shi X.M., Carrington J.C., Vance V.B. (1997). Plant viral synergism: The potyviral genome encodes a broad-range pathogenicity enhancer that transactivates replication of heterologous viruses. Plant Cell.

[24] Wu H.W., Lin S.S., Chen K.C., Yeh S.D., Chua N.H. (2010). Discriminating mutations of HC-Pro of Zucchini yellow mosaic virus with differential effects on small RNA pathways involved in viral pathogenicity and symptom development. Mol. Plant-Microbe Interact..

[25] Palauqui J.C., Elmayan T., Pollien J.M., Vaucheret H. (1997). Systemic acquired silencing: Transgene-specific post-transcriptional silencing is transmitted by grafting from silenced stocks to non-silenced scions. EMBO J..

[26] Voinnet O. (2005). Induction and suppression of RNA silencing: Insights from viral infections. Nat. Rev. Genet..

[27] Karjee S., Islam M.N., Mukherjee S.K. (2008). Screening and identification of virus-encoded RNA silencing suppressors. Methods Mol. Biol..

[28] Azzam O., Chancellor T.B. (2002). The biology; epidemiology; management of rice tungro disease in Asia. Plant Dis..

[29] Cabauatan P., Hibino H. (1985). Transmission of rice tungro bacilliform and spherical viruses by *Nephotettix virescens* (Distant). Philipp. Phytopathol..

[30] Jones M., Gough K., Dasgupta I., Rao B.S., Cliffe J., Qu R., Shen P., Kaniewska M., Blakebrough M., Davies J. (1991). Rice tungro disease is caused by an RNA and a DNA virus. J. Gen. Virol..

[31] Sanfaçon H., Wellink J., Le Gall O., Karasev A., van der Vlugt R., Wetzel T. (2009). *Secoviridae*: A proposed family of plant viruses within the order *Picornavirales* that combines the families *Sequiviridae* and *Comoviridae*; the unassigned genera *Cheravirus* and *Sadwavirus*; the proposed genus *Torradovirus*. Arch. Virol..

[32] Shen P., Kaniewska M., Smith C., Beachy R. (1993). Nucleotide sequence and genomic organization of rice tungro spherical virus. Virology.

[33] Hull R. (1996). Molecular biology of rice tungro viruses. Annu. Rev. Phytopathol..

[34] Mangrauthia S.K., Malathi P., Agarwal S., Sailaja B., Singh J., Ramkumar G., Krishnaveni D., Balachandran S.M. (2012). The molecular diversity and evolution of Rice tungro bacilliform virus from Indian perspective. Virus Genes.

[35] Kannan M., Saad M.M., Talip N., Baharum S.N., Bunawan H. (2019). Complete genome sequence of *Rice Tungro Bacilliform Virus* infecting asian rice (*Oryza sativa*) in Malaysia. Microbiol. Resour. Announc..

[36] Borah B.K., Sharma S., Kant R., Johnson A., Saigopal D.V.R., Dasgupta I. (2013). Bacilliform DNA-containing plant viruses in the tropics: Commonalities within a genetically diverse group. Mol. Plant Pathol..

[37] Dasgupta I., Hull R., Eastop S., Poggi-Pollini C., Blakebrough M., Boulton M.I., Davies J.W. (1991). Rice tungro bacilliform virus DNA independently infects rice after Agrobacterium-mediated transfer. J. Gen. Virol..

[38] Srilatha P., Yousuf F., Methre R., Vishnukiran T., Agarwal S., Poli Y., Raghurami Reddy M., Vidyasagar B., Shanker C., Krishnaveni D. (2019). Physical interaction of RTBV ORFI with D1 protein of *Oryza sativa* and Fe/Zn homeostasis play a key role in symptoms development during rice tungro disease to facilitate the insect mediated virus transmission. Virology.

[39] Tyagi H., Rajasubramaniam S., Rajam M.V., Dasgupta I. (2008). RNA-interference in rice against Rice tungro bacilliform virus results in its decreased accumulation in inoculated rice plants. Transgenic Res..

[40] Rajeswaran R., Golyaev V., Seguin J., Zvereva A.S., Farinelli L., Pooggin M.M. (2014). Interactions of Rice Tungro Bacilliform Pararetrovirus and Its Protein P4 with Plant RNA-Silencing Machinery. Mol. Plant-Microbe Interact..

[41] Mangrauthia S.K., Malathi P., Krishnaveni D., Reddy C.S., Viraktamath B.C., Balachandran S.M., Neeraja C.N., Biswal S.K. (2010). Rapid detection of rice tungro spherical virus by RT-PCR and dot-blot hybridization. J. Mycol. Plant Pathol..

[42] Malathi P., Mangrauthia S.K. (2013). Deciphering the multiplication behaviour of Rice tungro bacilliform virus by absolute quantitation through real-time PCR. Arch. Phytopathol. Plant Protect..

[43] Sparkes I.A., Runions J., Kearns A., Hawes C. (2006). Rapid; transient expression of fluorescent fusion proteins in tobacco plants and generation of stably transformed plants. Nat. Protoc..

[44] Das S.S., Sanan-Mishra N. (2014). Comparative analysis of RNAi suppression activity of proteins from two disparate viruses. Am. J. Plant Sci..

[45] Sambrook J., Fritsch E.F., Maniatis T. (1989). Molecular Cloning: A Laboratory Manual.

[46] Song X., Li P., Zhai J., Zhou M., Ma L., Liu B., Jeong D.H., Nakano M., Cao S., Liu C. (2012). Roles of DCL4 and DCL3b in rice phased small RNA biogenesis. Plant J..

[47] Wu Q., Wang X., Ding S.W. (2010). Viral suppressors of RNA-based viral immunity: Host targets. Cell Host Microbe.

[48] Wei L., Gu L., Song X., Cui X., Lu Z., Zhou M., Wang L., Hu F., Zhai J., Meyers B.C. (2014). Dicer-like 3 produces transposable element-associated 24-nt siRNAs that control agricultural traits in rice. Proc. Natl. Acad. Sci. USA.

[49] Llave C. (2010). Virus-derived small interfering RNAs at the core of plant–virus interactions. Trends Plant Sci..

[50] Pantaleo V. (2011). Plant RNA silencing in viral defence. Adv. Expt. Med. Biol..

[51] Rajeswaran R., Pooggin M.M. (2012). RDR6-mediated synthesis of complementary RNA is terminated by miRNA stably bound to template RNA. Nuc. Acids Res..

[52] Szittya G., Burgyán J. (2013). RNA interference-mediated intrinsic antiviral immunity in plants. Curr. Top. Microbiol. Immunol..

[53] Hull R. (2013). Plant Virology.

[54] Aregger M., Borah B.K., Seguin J., Rajeswaran R., Gubaeva E.G., Zvereva A.S., Windels D., Vazquez F., Blevins T., Farinelli L. (2012). Primary and secondary siRNAs in geminivirus-induced gene silencing. PLoS Pathog..

[55] Rajeswaran R., Aregger M., Zvereva A.S., Borah B.K., Gubaeva E.G., Pooggin M.M. (2012). Sequencing of RDR6-dependent double-stranded RNAs reveals novel features of plant siRNA biogenesis. Nuc. Acids Res..

[56] Blevins T., Rajeswaran R., Shivaprasad P.V., Beknazariants D., Si-Ammour A., Park H.S., Vazquez F., Robertson D., Meins F., Hohn T. (2006). Four plant Dicers mediate viral small RNA biogenesis and DNA virus induced silencing. Nuc. Acids Res..

[57] Hamilton A., Voinnet O., Chappell L., Baulcombe D. (2002). Two classes of short interfering RNA in RNA silencing. EMBO J..

[58] Himber C., Dunoyer P., Moissiard G., Ritzenthaler C., Voinnet O. (2003). Transitivity-dependent and independent cell-to-cell movement of RNA silencing. EMBO J..

[59] Dunoyer P., Schott G., Himber C., Meyer D., Takeda A., Carrington J.C., Voinnet O. (2010). Small RNA duplexes function as mobile silencing signals between plant cells. Science.

[60] Molnar A., Melnyk C.W., Bassett A., Hardcastle T.J., Dunn R., Baulcombe D.C. (2010). Small silencing RNAs in plants are mobile and direct epigenetic modification in recipient cells. Science.

[61] Fütterer J., Potrykus I., Brau M.P.V., Dasgupta I., Hull R., Hohn T. (1994). Splicing in a plant Pararetrovirus. Virology.

[62] Wang X.B., Jovel J., Udomporn P., Wang Y., Wu Q., Li W.X., Gasciolli V., Vaucheret H., Ding S.W. (2011). The 21-nucleotide; but not 22-nucleotide; viral secondary small interfering RNAs direct potent antiviral defense by two cooperative argonautes in *Arabidopsis thaliana*. Plant Cell.

[63] Blevins T., Rajeswaran R., Aregger M., Borah B.K., Schepetilnikov M., Baerlocher L., Farinelli L., Meins F., Hohn T., Pooggin M.M. (2011). Massive production of small RNAs from a non-coding region of Cauliflower mosaic virus in plant defense and viral counter-defense. Nuc. Acids Res..

[64] Takeda A., Sugiyama K., Nagano H., Mori M., Kaido M., Mise K., Tsuda S., Okuno T. (2002). Identification of a novel RNA silencing suppressor; NSs protein of Tomato spotted wilt virus. FEBS Letts..

[65] Fusaro A.F., Matthew L., Smith N.A., Curtin S.J., Dedic-Hagan J., Ellacott G.A., Watson J.M., Wang M.B., Brosnan C., Carroll B.J. (2006). RNA interference-inducing hairpin RNAs in plants act through the viral defence pathway. EMBO Rep..

[66] Mallory A.C., Vaucheret H. (2006). Functions of microRNAs and related small RNAs in plants. Nat. Genet..

[67] Moissiard G., Parizotto E.A., Himber C., Voinnet O. (2007). Transitivity in Arabidopsis can be primed; requires the redundant action of the antiviral Dicer-like 4 and Dicer-like 2, is compromised by viral-encoded suppressor proteins. RNA.

[68] Napoli C., Lemieux C., Jorgensen R. (1990). Introduction of a chimeric chalcone synthase gene into petunia results in reversible co-suppression of homologous genes in trans. Plant Cell.

[69] Di Serio F., Schöb H., Iglesias A., Tarina C., Bouldoires E., Meins F. (2001). Sense-and antisense-mediated gene silencing in tobacco is inhibited by the same viral suppressors and is associated with accumulation of small RNAs. Proc. Natl. Acad. Sci. USA.

[70] Zvereva A.S., Pooggin M.M. (2012). Silencing and innate immunity in plant defense against viral and non-viral pathogens. Viruses.

[71] Malathi P., Muzammil S.A., Krishnaveni D., Balachandran S.M., Mangrauthia S.K. (2019). Coat protein 3 of Rice tungro spherical virus is the key target gene for development of RNAi mediated tungro disease resistance in rice. Agri Gene.

[72] Mangrauthia S.K., Malathi P., Balachandran S.M., Reddy C.S., Viraktamath B.C. (2010). Global analysis of Rice tungro spherical virus coat proteins reveals new roles in evolutionary consequences. J. Plant Biochem. Biotechnol..

