# Supplementary material for: Coordinated Action of RTBV and RTSV Proteins Suppress Host RNA Silencing Machinery
Source: Microorganisms. 2022 Jan 18;10(2):197. doi: 10.3390/microorganisms10020197 (PMC8875415; doi:10.3390/microorganisms10020197)
Supplement: Supplementary file 1 [file microorganisms-10-00197-s001.zip › microorganisms-1524413-supplementary-final.pdf]

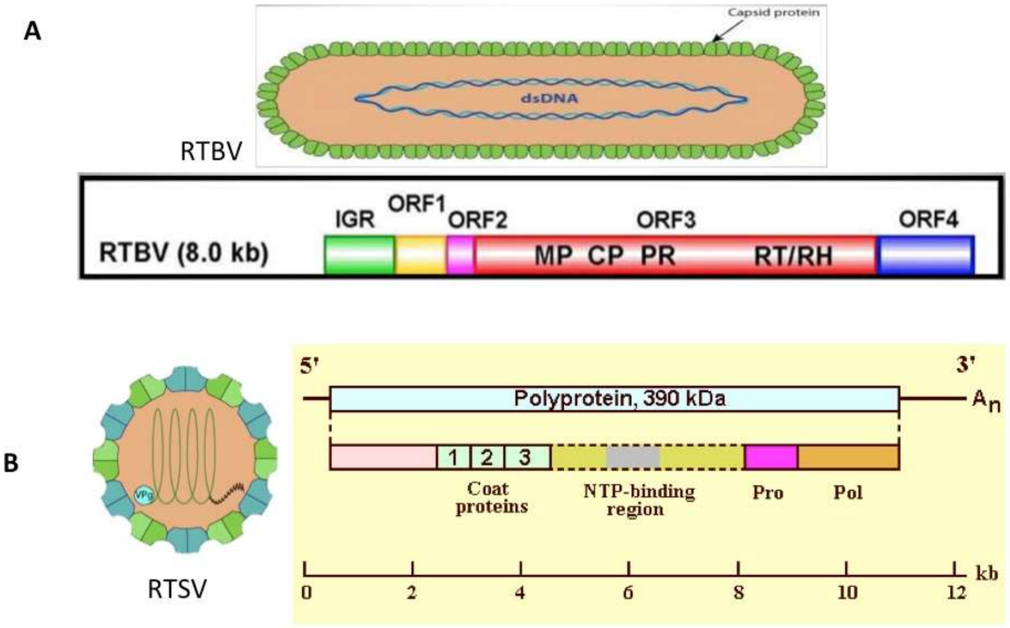

(SUPPLEMENTARY)
FIGURE S1

Figure S1: Genomic organization and structure of RTBV and RTSV.

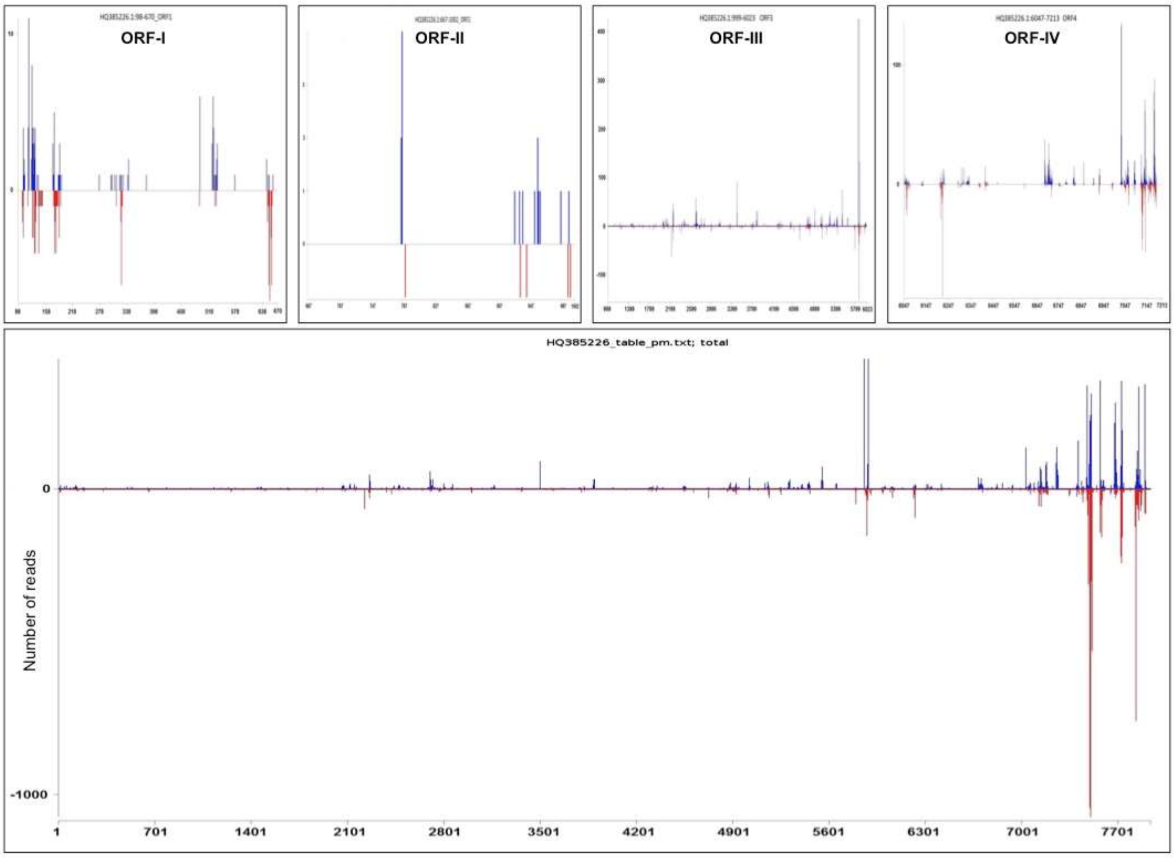

FIGURE S2

Figure S2: Plot of siRNA mol-ecules mapping to different positions on both the strands of the RTBV genome. The boxes on top show the mapping pattern on individual RTBV ORFs. The nucleotide positions are indicated on the scale in each figure. The siRNAs mapping to positive strand of the ORF are represented by blue lines while those mapping to the negative strand are represented by red lines.

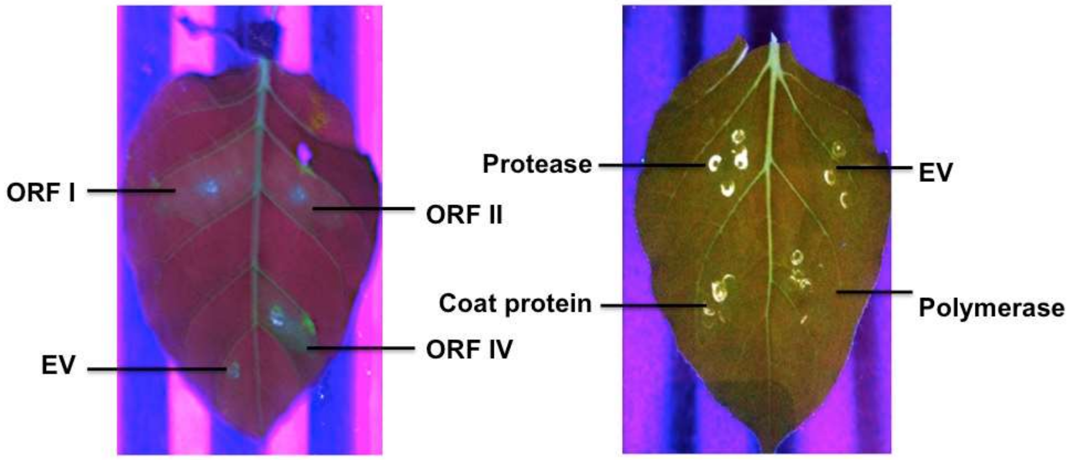

(SUPPLEMENTARY)
FIGURE S3

Figure S3: In-dividual infiltrations to assay the in planta reversal of GFP silencing activity by different RTBV and RTSV ORFs. The RTBV ORF-I, ORF-II and the RTSV ORFs encoding protease, polymerase and coat protein 3 did not show a clear suppressor activity. The region infiltrated with empty vector (EV) served as control.

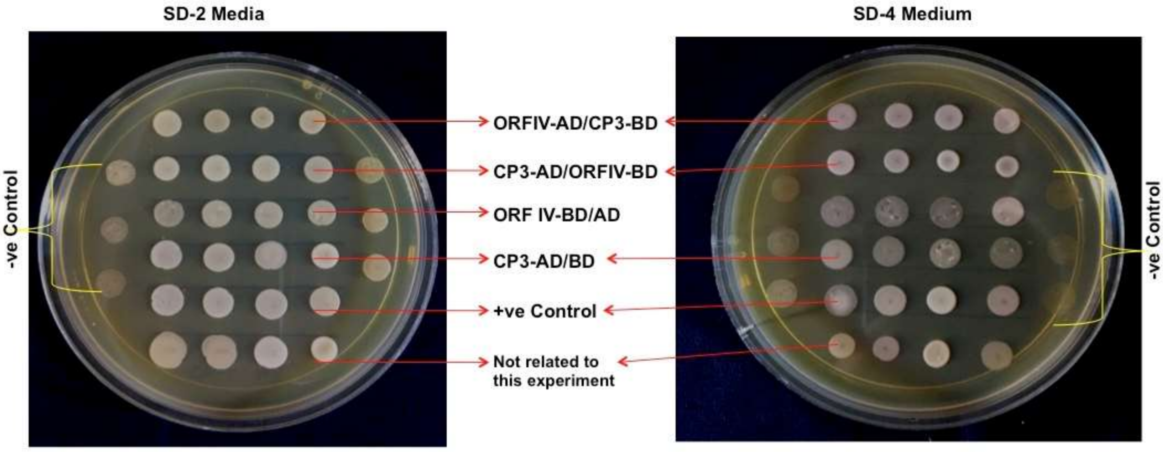

(SUPPLEMENTARY)
FIGURE S2

(SUPPLEMENTARY)
FIGURE S4

Figure S4: Plates showing the yeast two-hybrid interaction of pGAD-CP3 and pGBD-ORF-IV. Yeast colonies were co-transformed and selected on (A) two drop out medium (Leu ¯ Trp ¯) and (ii) four drop out medium (Leu ¯ Trp ¯ His ¯ , Ade-). Co-infiltration of pGAD-CP3 with pGBD and pGAD with pGBD-ORF-IV was performed as control. The positive control was same as that provided in the kit. For negative control empty AD and BD vectors were co-transformed.
